# Dorsal longitudinal foreskin cut is associated with reduced risk of HIV, syphilis and genital herpes in men: a cross-sectional study in Papua New Guinea

**DOI:** 10.7448/IAS.20.01/21358

**Published:** 2017-04-03

**Authors:** Andrew J Vallely, David MacLaren, Matthew David, Pamela Toliman, Angela Kelly-Hanku, Ben Toto, Rachael Tommbe, Zure Kombati, Petronia Kaima, Kelwyn Browne, Clement Manineng, Lalen Simeon, Claire Ryan, Handan Wand, Peter Hill, Greg Law, Peter M Siba, W John H McBride, John M Kaldor

**Affiliations:** ^a^ Public Health Intervention Research Group, Kirby Institute, UNSW Australia, Sydney, Australia; ^b^ Sexual and Reproductive Health Research Unit, Papua New Guinea Institute of Medical Research, Goroka, Papua New Guinea; ^c^ College of Medicine and Dentistry, James Cook University, Cairns, Australia; ^d^ School of Health Science, Pacific Adventist University, Port Moresby, Papua New Guinea; ^e^ Department of Pathology, Tininga Clinic, Mt Hagen General Hospital, Papua New Guinea; ^f^ Sexual Health and HIV/AIDS Prevention and Control, National Department of Health, Port Moresby, Papua New Guinea; ^g^ Centre for International Health, The Burnet Institute, Melbourne, Australia; ^h^ School of Public Health, University of Queensland, Brisbane, Australia

**Keywords:** male genital cutting, superincision, Pacific

## Abstract

**Introduction**: Various forms of penile foreskin cutting are practised in Papua New Guinea. In the context of an ecological association observed between HIV infection and the dorsal longitudinal foreskin cut, we undertook an investigation of this relationship at the individual level.

**Methods**: We conducted a cross-sectional study among men attending voluntary confidential HIV counselling and testing clinics. Following informed consent, participants had a face-to-face interview and an examination to categorize foreskin status. HIV testing was conducted on site and relevant specimens collected for laboratory-based Herpes simplex type-2 (HSV-2), syphilis, Chlamydia trachomatis (CT), *Neisseria gonorrhoeae* (NG), and *Trichomonas vaginalis* (TV) testing.

**Results**: Overall, 1073 men were enrolled: 646 (60.2%) were uncut; 339 (31.6%) had a full dorsal longitudinal cut; 72 (6.7%) a partial dorsal longitudinal cut; and 14 (1.3%) were circumcised. Overall, the prevalence of HIV was 12.3%; HSV-2, 33.6%; active syphilis, 12.1%; CT, 13.4%; NG, 14.1%; and TV 7.6%. Compared with uncut men, men with a full dorsal longitudinal cut were significantly less likely to have HIV (adjusted odds ratio [adjOR] 0.25, 95%CI: 0.12, 0.51); HSV-2 (adjOR 0.60, 95%CI: 0.41, 0.87); or active syphilis (adjOR 0.55, 95%CI: 0.31, 0.96). This apparent protective effect was restricted to men cut prior to sexual debut. There was no difference between cut and uncut men for CT, NG or TV.

**Conclusions**: In this large cross-sectional study, men with a dorsal longitudinal foreskin cut were significantly less likely to have HIV, HSV-2 and syphilis compared with uncut men, despite still having a complete (albeit morphologically altered) foreskin. The protective effect of the dorsal cut suggests that the mechanism by which male circumcision works is not simply due to the removal of the inner foreskin and its more easily accessible HIV target cells. Exposure of the penile glans and inner foreskin appear to be key mechanisms by which male circumcision confers protection.

Further research in this unique setting will help improve our understanding of the fundamental immunohistologic mechanisms by which male circumcision provides protection, and may lead to new biomedical prevention strategies at the mucosal level.

## Introduction

Papua New Guinea (PNG) has among the highest prevalences of HIV, syphilis and other sexually transmitted infections (STIs) of any Asia-Pacific country [1], with an estimated adult HIV prevalence of 0.6–0.8% and significant variations by geographical region and in different sub-populations [2,3]. PNG is a geographically, linguistically and culturally diverse nation with a wide variety of socio-cultural beliefs and practices. Across communities, there is a range of traditional and contemporary penile modification practices [4–7]. These include the full removal of the foreskin (male circumcision); full and partial dorsal longitudinal foreskin cuts (“long” or “straight” cut, and the “V” cut, respectively); insertion of objects into the skin of the foreskin and penile shaft; injection of oil and other fluids along the penile shaft; and/or a combination of these practices [4–7].

**Figure 1.**
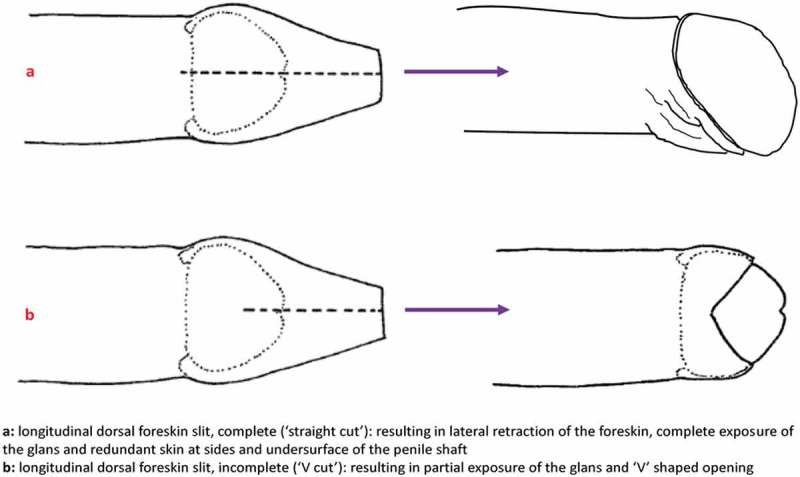
Full (complete) and partial (incomplete) foreskin cuts.

We found that the most common modification was the dorsal longitudinal foreskin cut, which in its most extensive form (a “full” cut to the base of the glans penis) resulted in 100% exposure of the glans and inner foreskin, with an appearance closely resembling that of male circumcision (Figure 1) [4,6]. We have postulated that this type of cut, like circumcision, could confer protection against acquisition of HIV and certain other STIs [8–11] as it results in lateral retraction and eversion of the foreskin [4,6]. This hypothesis is supported by the geographic association between high prevalence of this type of foreskin cut and lower prevalence of HIV infection within PNG [12]. It was this type of ecological association observed in Africa which first signalled the possibility, subsequently proven in large-scale clinical trials, that male circumcision might be protective against HIV and other infections in Africa [13,14].

In order to determine whether foreskin cutting as practised in PNG may indeed be protective against HIV and other STIs, we conducted a cross-sectional study among men attending voluntary confidential HIV counselling and testing clinics.

## Methods

### Study design and participants

All men aged 18 years and over attending voluntary confidential HIV counselling and testing clinics in the Highlands region of PNG during the period 1 October 2013–30 June 2015 were invited to participate in a cross-sectional study. Six clinics accredited by the National Department of Health (NDoH) located in Enga (2 clinics), Simbu (2), Jiwaka (1) and Western Highlands (1) Provinces were selected as study sites based on the quality of their HIV testing and counselling services (as recognized by the PNG NDoH); client attendance figures; provincial and clinic-level interest in study participation; and earlier surveillance and research data indicating relatively high prevalences of both HIV infection and penile foreskin cutting in these settings [3,6,12].

### Study procedures

Men were provided with information about the study whilst waiting to undergo pre-test counselling, and if willing to participate, asked to complete written informed consent procedures in accordance with international guidelines [15]. Participants were enrolled consecutively and each assigned a unique alphanumeric study ID number that was used to identify clinical and laboratory records.

A trained male nurse-counsellor carried out a face-to-face interview in which socio-demographic, sexual behavioural and clinical information were collected. A clinical examination was conducted to verify and categorize penile foreskin cutting status according to the type of cut and the degree of exposure of the glans penis. The quality of clinical examination and the verification/categorisation process was monitored throughout by the study coordinator (MD) who directly observed clinical examinations conducted by research staff at all clinic sites on the days of his monthly site monitoring and supervision visits.

Men provided venepuncture and urine specimens for laboratory-based STI testing before proceeding to pre-test counselling and onsite HIV testing, conducted in accordance with PNG national guidelines. Men found to be HIV positive were provided with post-test counselling and referral for additional services and support, as per PNG national guidelines. Urine and serum specimens were stored onsite at −20°C until transfer to the PNG Institute of Medical Research (PNGIMR) in Goroka for testing. Interview, examination and HIV test result data were entered into study-specific case record forms (CRFs) that contained no unique subject identifiers or locator information and which were stored securely in locked cabinets at each clinic site.

Participants with clinical features suggestive of an STI were managed according to national STI syndromic management guidelines. All participants were advised to re-attend clinic 4–6 weeks after enrolment in order to receive the results of their laboratory investigations and further treatment as indicated.

### Laboratory methods

HIV testing was conducted using two simple rapid tests at point-of-care in accordance with PNG national guidelines: Determine HIV-1/2 (Alere, Hannover, Germany) followed by confirmatory Stat-Pak HIV 1/2 (Chembio, Medford NY, USA). HIV testing was carried out as per manufacturers’ instructions by an accredited HIV counsellor at each site, and their practice directly observed by the study coordinator (MD) throughout the study period during monthly site monitoring and supervision visits. All clinic sites were enrolled in the PNG National Department of Health quality assurance programme for HIV rapid testing. Men who had both a reactive Determine test and a reactive Stat-Pak test were considered to be HIV positive and were referred to local HIV treatment and care services, as per PNG national guidelines.

All other laboratory investigations were conducted at the PNGIMR HIV/STI Research Laboratory in Goroka. Syphilis testing used a single rapid point-of-care screening test (SD Bioline anti-TP 3.0, Alere, Hannover, Germany) followed by a confirmatory laboratory-based rapid plasma reagin (RPR) test. Those with a positive anti-TP plus a positive RPR test (any titre) were considered to have active syphilis. Testing for *Herpes simplex* virus type-2 (HSV-2) was performed on serum samples using a standard IgG-specific ELISA (Kalon Biologicals, Aldershot, UK) according to the manufacturer’s instructions and using the recommended optical density ratios for the interpretation of results. Urine specimens were tested for *C. trachomatis, N. gonorrhoeae* and *T. vaginalis* by real-time polymerase chain reaction (PCR) using established methods, primers and probes, and performed using a Bio-Rad CFX 96 machine and CFX Manager Software (version 2.1) as previously described [7].

The laboratory was enrolled in an external quality assurance programme through the Royal College of Pathologists of Australia for syphilis and HSV-2 serology and for *N. gonorrhoeae* and *C. trachomatis* PCR. There was no available programme for *T. vaginalis* PCR.

### Data management

Participant study folders (containing completed case record forms and laboratory results slips) were subject to quarterly audits by the study coordinator (MD) throughout. Data were entered into a study-specific MS Access database at a central data management facility in Goroka. All database entries were validated against individual participant study folders for accuracy by the study coordinator at study mid-point (n = 524) and completion (n = 1073). Inconsistencies between the electronic database and source data were noted in a separate electronic Error Log and underwent review and resolution. Laboratory test results were entered into written test logs prior to entry into a study-specific laboratory MS Excel database by a dedicated laboratory graduate scientific officer (BT). All database and written log entries were checked for consistency by the Head of the HIV/STI Research Laboratory (PT) weekly during the study and prior to results slips being printed for the communication of results to clinic staff. On study completion, the final validated clinical and laboratory databases were locked by the study statistician (HW) prior to statistical analyses.

### Statistical analyses

Descriptive statistics, including percentages and medians with interquartile ranges (IQR) for categorical and continuous variables, respectively, were used to describe the study population overall and by HIV status. Logistic regression was used to examine associations between penile cutting status, HIV infection and other STI outcome variables; and to adjust for potential confounding variables including sexual behaviour. The primary objective of this part of the analysis was to adjust the results for potentially important risk factors and/or confounders identified in univariate analysis, rather than to identify independent correlates of infection. Two-sided hypotheses and tests were adopted for all statistical inferences and p values <0.05 were considered statistically significant. All analyses were performed using Stata V.14.0 (College Station, TX, USA).

### Sample size and power

We estimated that a sample size of around 1300 men would be required to detect a reduction in risk associated with full dorsal longitudinal cut of 0.6 or greater with 80% power at the 5% significance level in a population where the prevalence of HIV in uncut men is around 12% and the prevalence of dorsal longitudinal cut around 40% [6,7,12].

### Ethical considerations

Following written local-level endorsement of the protocol by Provincial AIDS Committees, ethical approval was provided by the Research Advisory Committee of the National AIDS Council Secretariat (RAC No. 12.008), the Medical Research Advisory Committee of the National Department of Health (MRAC No. 12.24), and the Institutional Review Board of the PNG Institute of Medical Research (IRB No. 1224) in PNG. Ethics approval was also obtained from the Human Research Ethics Committee of the University of New South Wales in Australia (HREC No. 13009). Written informed consent (signature or witnessed thumbprint) was obtained from all participants prior to enrolment.

## Results

A total of 1,073 men were enrolled at six HIV testing sites in four provinces. Around 90% of all those invited to participate agreed to take part in the study. The prevalence of HIV infection was 12.3% (132/1073) (Table 1). Men found to have HIV reported a younger median age of sexual debut compared with HIV negative men (19 years *vs*. 20 years; p = 0.004), and were more likely to be separated, divorced or widowed. No significant differences were observed in median age, province of birth, educational attainment, employment status, religion or sexual behavioural characteristics between HIV-positive and -negative men.

**Table 1.** Association between HIV infection and selected socio-demographic and behavioural characteristics

	All men *n* = 1073 *N* (%)	HIV negative men *n* = 941 *N* (%)	HIV positive men *n* = 132 *N* (%)	Odds Ratio (95% CI)	*p*-value
**Socio-demographic characteristics**					
Median age (IQR), years	30 (25.38)	30 (25.38)	32 (26.40)	–	0.183^§^
Age group, years					
18–24	222 (20.69)	197 (20.94)	25 (18.94)	1	
25–29	254 (23.67)	223 (23.70)	31 (23.48)	1.10 (0.63,1.92)	0.750
30–34	166 (15.47)	146 (15.52)	20 (15.15)	1.10 (0.58,2.02)	0.811
35–39	176 (16.40)	156 (16.58)	20 (15.15)	1.01 (0.54,1.89)	0.974
40–44	114 (10.62)	99 (10.52)	15 (11.36)	1.20 (0.60,2.37)	0.612
45+	141 (13.14)	120 (12.75)	21 (15.91)	1.38 (0.74,2.57)	0.312
**Marital status**					
Married	713 (66.45)	625 (66.42)	88 (66.67)	1	
Single	274 (25.54)	248 (26.35)	26 (19.70)	0.74 (0.47,1.18)	0.210
Separated/divorced/widowed	86 (8.01)	68 (7.23)	18 (13.64)	1.88 (1.07,3.31)	**0.029**
**Province of birth**				<0.001	
Enga	630 (58.71)	563 (59.83)	67 (50.76)		
Simbu	184 (17.15)	169 (17.96)	15 (11.36)		
Western Highlands	98 (9.13)	76 (8.08	22 (16.67)		
Jiwaka	43 (4.01)	35 (3.72)	8 (6.06)		
Other	118 (11.00)	98 (10.41)	20 (15.15)		
**Province where currently live**				<0.001	
Enga	643 (59.93)	581 (61.74)	62 (46.97)		
Simbu	185 (17.24)	170 (18.07)	15 (11.36)		
Western Highlands	153 (14.26)	116 (12.33)	37 (28.03)		
Jiwaka	57 (5.31)	44 (4.68)	13 (9.85)		
Other	35 (3.26)	30 (3.18)	5 (3.79)		
**Employment**					
Not employed	841 (78.38)	735 (78.11)	106 (80.30)	1	
Yes employed	232 (21.62)	206 (21.86)	26 (19.70)	0.88 (0.55,1.38)	0.566
Teacher	37 (15.95)	34 (16.50)	3 (11.54)	–	
Security	38 (16.38)	34 (16.50)	4 (15.38)		
House duties	3 (1.29)	3 (1.46)	–	–	
Farmer	13 (5.60)	12 (5.83)	1 (3.85)	–	
Other	106 (45.69)	90 (43.69)	16 (61.54)	–	
**Religion**					
Catholic	269 (25.07)	238 (25.29)	31 (23.48)	1	
Lutheran	199 (18.55)	171 (18.17)	28 (21.21)	1.26 (0.73,2.17)	0.413
Seventh Day Adventist	242 (22.55)	219 (23.27)	23 (17.42)	0.81 (0.46,1.43)	0.459
Pentecostal	178 (16.59)	161 (17.11)	17 (12.88)	0.81 (0.43,1.51)	0.510
Other	185 (17.24)	152 (16.15)	33 (25.00)	1.67 (0.98,2.83)	0.059
**Education**					
No education	281 (26.19)	249 (26.46)	32 (24.24)	1	
Primary school	473 (44.08)	399 (42.40)	74 (56.06)	1.44 (0.93,2.25)	0.105
Secondary school	217 (20.22)	199 (21.15)	18 (13.64)	0.70 (0.38,1.29)	0.257
Other	102 (9.51)	94 (9.99)	8 (6.06)	0.66 (0.29,1.49)	0.319
**Sexual behavioural characteristics**					
Ever had vaginal sex	1065 (99.25)	933 (99.15)	132 (100.00)	–	
Median age (IQR) at first vaginal sex, years	20 (18,23)	20 (18,23)	19 (17,22)		**0.004**^§^
**Age at sexual debut**					
18 years or less	723 (67.38)	649 (68.07)	74 (56.06)	1	
19 years or older	350 (32.62)	292 (31.03)	58 (43.94)	1,74 (1.20, 2.52)	**0.003**
**Partner at last vaginal sex**					
Regular partner/spouse	515 (48.00)	447 (47.50)	68 (51.52)	1	
Casual partner	522 (48.65)	463 (49.20)	59 (44.70)	0.84 (0.58, 1.22)	0.351
Sex worker	28 (2.61)	23 (2.44)	5 (3.79)	1.43 (0.53, 3.89)	0.484
Other	8 (0.75)	8 (0.85)	–	–	–
**Condom use at last vaginal sex**					
Yes	130 (12.12)	118 (12.54)	12 (9.09)	0.70 (0.37, 1.30)	0.258
**Condom use in last month during vaginal sex**					
Always	31 (2.89)	29 (3.08)	2 (1.52)	1	
Sometimes	423 (39.42)	376 (39.96)	47 (35.61)	1.81 (0.42, 7.84)	0.426
Never	619 (57.69)	536 (56.96)	83 (62.88)	2.25 (0.53, 9.59)	0.275
**Number of partners had vaginal sex with in the last month**					
0 or 1	641 (59.74)	569 (60.47)	72 (54.55)	1	
2	230 (21.44)	203 (21.57)	27 (20.45)	1.05 (0.66, 1.68)	0.835
3 +	202 (18.83)	169 (17.96)	33 (25.00)	1.54 (0.99, 2.41)	0.057
**Ever had anal sex**	63 (5.87)	53 (5.63)	10 (7.58)	1.37 (0.68, 2.77)	0.377
Ever had anal sex with a woman	57 (98.28)	48 (97.96)	9 (100.00)	–	
Ever had anal sex with a man	1 (1.72)	1 (2.04)	–	–	
Condom use at last anal sex	13 (21.67)	12 (23.53)	1 (11.11)	–	
**Condom use in last month during anal sex**					
Always	5 (8.33)	4 (7.84)	1 (11.11)	–	
Sometimes	10 (16.67)	10 (19.61)	–	–	
Never	45 (75.00)	37 (72.55)	8 (88.89)	–	

^§^ Rank sum test.

Overall, 60.2% of those enrolled had an uncut foreskin; 31.6% had a full dorsal longitudinal cut; 6.7% a partial dorsal longitudinal cut; and 1.3% were circumcised (Table 2). The prevalence of the full dorsal longitudinal cut was significantly higher among men born in Simbu (80/184; 43.5%) and Jiwaka (18/43; 41.9%) compared with men born in Western Highlands (28/98; 28.6%) and Enga (168/630; 26.7%) (data not shown). The prevalence of HSV-2 was 33.6%; active syphilis, 12.1%; *C. trachomatis*, 13.4%; *N. gonorrhoeae*, 14.1%; and *T. vaginalis*, 7.6%. HIV-positive men were significantly more likely to be uncut, to have active syphilis, *C. trachomatis* or HSV-2 infection, compared with HIV-negative men, but there was no difference between HIV-positive and -negative men in reported genital symptoms at enrolment (Table 2). Men with a full dorsal longitudinal cut were significantly less likely to have HIV infection than uncut or partially cut men.

**Table 2.** Association between HIV infection, clinical and laboratory characteristics

	All men *n* = 1073 *N* (%)	HIV negative men *n* = 941 *N* (%)	HIV positive men *n* = 132 *N* (%)	Odds Ratio (95% CI)	*p*-value
**Penile foreskin status^1^**					
Uncut	646 (60.21)	549 (58.34)	97 (73.48)	1	
Incomplete dorsal longitudinal cut	72 (6.71)	63 (6.70)	9 (6.82)	0.81 (0.39, 1.96)	0.575
Complete dorsal longitudinal cut	339 (31.59)	316 (33.58)	23 (17.42)	0.41 (0.26, 0.66)	**<0.001**
Male circumcision	14 (1.30)	11 (1.17)	3 (2.27)	1.55 (0.42, 5.65)	0.508
**Other penile modifications^1^**					
Penile insert present	8 (0.75)	8 (0.85)	–	–	
Penile injection present	45 (4.19)	38 (4.04)	7 (5.30)	1.33 (0.58, 3.04)	0.499
**Current symptoms suggestive of an STI**					
Abdominal pain	108 (10.07)	91 (9.67)	17 (12.88)	1.38 (0.79, 2.40)	0.253
Lumps in groin	28 (2.61)	24 (2.55)	4 (3.03)	1.19 (0.41, 3.50)	0.746
Pain on passing urine	139 (12.95)	128 (13.60)	11 (8.33)	0.58 (0.30, 1.10)	0.095
Penile discharge	86 (8.01)	78 (8.29)	8 (6.06)	0.71 (0.34, 1.51)	0.379
Genital sore	22 (2.05)	20 (2.13)	2 (1.52)	0.70 (0.16, 3.07)	0.645
Scrotal itching	26 (2.42)	23 (2.44)	3 (2.27)	0.93 (0.27, 3.14)	0.905
Anal itching	13 (1.21)	9 (0.96)	4 (3.03)	3.24 (0.98, 10.66)	0.054
**Laboratory findings**					
Active syphilis (anti-TP+/RPR+, any titre)	130 (12.12)	105 (11.16)	25 (18.94)	1.86 (1.15, 3.01)	**0.011**
*C. trachomatis*	144 (13.42)	118 (12.54)	26 (19.70)	1.71 (1.10, 2.74)	**0.025**
HSV-2	357 (33.57)	278 (29.54)	79 (59.85)	3.55 (2.44, 5.17)	**<0.001**
*N. gonorrhoeae*	152 (14.17)	131 (13.92)	21 (15.91)	1.7 (0.71, 1.93)	0.540
*T. vaginalis*	81 (7.55)	73 (7.76)	8 (6.06)	0.77 (0.36, 1.63)	0.491
1 or more of CT, NG, TV	311 (28.98)	264 (28.06)	47 (35.61)	1.42 (0.97, 2.08)	0.074
2 or more of CT, NG, TV	59 (5.50)	52 (5.53)	7 (5.30)	0.96 (0.43, 2.15)	0.916

^1^ Categorised according to clinical examination findings.

After adjusting for marital status, province of birth, province of current residence, age of sexual debut, and number of sex partners in the previous month, foreskin status was independently associated with HIV, HSV-2 and syphilis (Table 3). Men with a full dorsal longitudinal cut were significantly less likely than uncut men to have HIV (adjusted odds ratio [adjOR] 0.25, 95% CI: 0.12, 0.51); HSV-2 (adjOR 0.60, 95% CI: 0.41, 0.87); or active syphilis (adjOR 0.55, 95% CI: 0.31, 0.96). There was no difference between cut and uncut men for *C. trachomatis, N. gonorrhoeae* or *T. vaginalis* infection. The association between full dorsal longitudinal cut and lower prevalence of HIV and HSV-2 was restricted to men who reported being cut prior to sexual debut (Table 4).

**Table 3.** Association between penile foreskin cutting, HIV and sexually transmitted infections

	Uncut *n* = 646 *N* (%)	Complete dorsal longitudinal cut *n* = 339 *N* (%)	Odds Ratio (95% CI)	*p*-value	Adjusted^1^ Odds Ratio (95% CI)	*p*-value
HIV+	97 (15.02)	23 (6.78)	0.41 (0.26, 0.66)	<0.001	0.25 (0.12, 0.51)	**<0.001**
HSV-2	247 (38.24)	90 (26.55)	0.58 (0.44, 0.78)	<0.001	0.60 (0.41, 0.87)	**0.007**
Active syphilis (anti-TP+/RPR+)	99 (15.33)	23 (6.78)	0.40 (0.25, 0.65)	<0.001	0.55 (0.31, 0.96)	**0.037**
*C. trachomatis*	83 (12.85)	47 (13.86)	1.10 (0.74, 1.60)	0.55	0.93 (0.58, 1.50)	0.768
*N. gonorrhoeae*	92 (14.24)	49 (14.45)	1.02 (0.70, 1.48)	0.928	0.79 (0.49, 1.28)	0.332
*T. vaginalis*	56 (8.67)	25 (7.37)	0.84 (0.51, 1.37)	0.483	0.98 (0.55, 1.73)	0.932
1 or more of HIV, syphilis, HSV-2	314 (48.61)	106 (31.27)	0.48 (0.36, 0.63)	<0.001	0.51 (0.36, 0.73)	**<0.001**
1 or more of CT, NG, TV	197 (30.50)	93 (27.43)	0.86 (0.64, 1.15)	0.317	0.73 (0.51, 1.05)	0.089
2 or more of CT, NG, TV	30 (4.64)	25 (7.37)	1.63 (0.95, 2.83)	0.079	1.40 (0.72, 2.75)	0.322

^1^ Adjusted for number of sex partners, age at sexual debut, marital status, province of birth, province of residence.

**Table 4.** Association between HIV, HSV-2, active syphilis and timing of dorsal longitudinal cut^1^

HIV	*N*	Odds ratio (95% CI)	*p*-Value	Adjusted^2^ odds ratio (95% CI)	*p*-Value
Uncut	646	1		1	
Dorsal cut before sexual debut	150	0.24 (0.10, 0.55)	***p* = 0.001**	0.20 (0.08, 0.48)	***p* < 0.001**
Dorsal cut after sexual debut	128	0.70 (0.38, 1.26)	*p* = 0.231	0.61 (0.32, 1.14)	*p* = 0.122
**HSV-2**	**N**	**Odds ratio** (95% CI)	***p*-Value**	**Adjusted^2^ odds ratio** (95% CI)	***p*-Value**
Uncut	646	1		1	
Dorsal cut before sexual debut	150	0.40 (0.26, 0.62)	***p* < 0.001**	0.50 (0.32, 0.78)	***p* = 0.002**
Dorsal cut after sexual debut	128	0.73 (0.49, 1.10)	p = 0.136	0.85 (0.55, 1,30)	p = 0.449
**Active syphilis** (anti-TP+/RPR+)	**N**	**Odds ratio** (95% CI)	***p*-Value**	**Adjusted^2^ odds ratio** (95% CI)	***p*-Value**
Uncut	646	1		1	
Dorsal cut before sexual debut	150	0.35 (0.17, 0.72)	***p* = 0.004**	0.44 (0.21, 0.91)	***p* = 0.028**
Dorsal cut after sexual debut	128	0.32 (0.14, 0.71)	***p* = 0.005**	0.37 (0.17, 0.83)	***p* = 0.015**

^1^ Data on timing of cut in relation to age at sexual debut were available on 278 of 339 men with a full dorsal longitudinal cut (82.0%), among whom 150//278 (54.0%) reported cutting prior to sexual debut and 128/278 (46.0%) reported cutting after age of sexual debut.

^2^ Adjusted for number of sex partners, marital status, current province.

Among 427 men with any type of foreskin cut, 83% (353/427) provided reasons for cutting, the most frequent being to improve genital hygiene (44.8%); to increase partners’ sexual pleasure (32.6%); to increase own sexual pleasure (30.6%); and to prevent STIs (25.2%; data not shown). Compared with HIV-negative men (327/353), HIV positive men (26/353) were significantly less likely to report cutting to increase their own sexual pleasure (3.6% *vs*. 32.7%; p = 0.002) and more likely to state that cutting was done to prevent infections (42.3% *vs*. 23.9%; p = 0.037).

## Discussion

We undertook the world’s first analytical investigation of the relationship between a distinct form of penile foreskin cutting and sexually transmitted infections, including HIV. We found that men with a full dorsal longitudinal foreskin cut were significantly less likely to have HIV, HSV-2 or active syphilis compared with uncut men. This association persisted after adjusting for socio-demographic and sexual behavioural risk factors. There was no difference between cut and uncut men in regards *C. trachomatis, N. gonorrhoeae* or *T. vaginalis* infection.

Our findings are consistent with earlier research indicating the potential protective effect of male circumcision against HIV, HSV-2 and syphilis (that typically infect the inner foreskin and glans) [16–18], but not against *C. trachomatis, N. gonorrhoeae* and *T. vaginalis* (that are primarily urethral infections) [19,20]. It should also be noted, however, that such associations have not been universally reported [20–23].

We consider alternative explanations for our principal finding unlikely due to the strategies used to minimize bias. Genital examination to confirm foreskin status was conducted prior to onsite HIV testing so that if there was any misclassification error it was not associated with HIV, as status was unknown at the time of examination. As noted above, the quality of clinical examinations conducted and the categorisation of participant foreskin status was reviewed monthly by the in-country clinical coordinator (MD) and so we are confident in the accuracy of group allocation. We considered it unethical to conduct HIV testing prior to examination because this would have resulted in undue distress for men newly diagnosed as having HIV, in addition to introducing selection bias due to the differential enrolment of HIV positive and negative individuals. We followed standard national HIV testing guidelines and quality assurance procedures, which did not incorporate strategies for identifying men with very recent infection, who may not have had detectable HIV antibodies. This may have led to some degree of misclassification error but there is no reason to assume that this was differential and therefore the only effect may have been to underestimate the true association between the dorsal longitudinal cut and HIV infection. Similarly, there is no reason to assume any differential misclassification of other STIs. It is possible that men who perceived themselves to be at increased risk of HIV and STIs may have undergone cutting to provide protection against infection. If cuts were carried out after individuals had already acquired HIV, this would then have led to an underestimation of the association between the dorsal cut and HIV infection.

Our finding of a protective effect against HIV and HSV-2 was restricted to men who reported having a full dorsal cut before sexual debut, adding weight to the inference that the cut is protective. The findings of the current study are also consistent with our earlier research in this setting in which we found a geographic association between the dorsal longitudinal cut and HIV infection [12]; and a lower incidence of syphilis among men with this type of cut compared with uncut men (relative risk: 0.10; 95% CI: 0.01, 1.84; p = 0.032) [7]. The timeframe between performing a dorsal cut and the start of its apparent protective effect remains unclear, however. Earlier research suggests a variable time period of several months to years during which a flap of foreskin hangs below the glans prior to skin atrophy and retraction resulting in an appearance closely resembling male circumcision [4,5]. This provides challenges to policy-makers and programme managers. Sexually active men undergoing dorsal longitudinal cuts may be at temporary heightened risk during the healing period of the fresh wound. There then appears to be a variable time period during which the protective effect of the dorsal cut is established. If dorsal longitudinal cuts continue to be conducted outside the formal health sector, men will not receive appropriate risk reduction counselling, health education and condom provision during these periods of heightened or uncertain HIV acquisition risk. Failing to address these issues could undermine any potential public health benefits of the dorsal cut in this setting.

Although traditional forms of male circumcision involving the full removal of the foreskin have been practised since ancient times [24–26], and remain comparatively widespread globally, the dorsal longitudinal foreskin cut (often referred to as “superincision” in the anthropological literature) has only been reported in a small number of societies worldwide, principally as part of male initiation rituals in parts of Polynesia [27–30], Solomon Islands [29], PNG [5,29,31,32], and western Borneo [33]. The dorsal longitudinal cut has become widespread in contemporary PNG society [4,6] including in communities that did not conduct genital cutting as part of male initiation in the pre-colonial era; and is now conducted secretly, without associated ritual or ceremony, and appears driven by a perception that it leads to greater sexual pleasure and improved genital hygiene [5,6].

Our findings have a number of implications beyond PNG. First, the apparent protective effect of the dorsal longitudinal cut and the lack of protection conferred by the partial cut provide a unique opportunity to improve our understanding of the fundamental mechanisms by which male circumcision protects men against HIV acquisition and other STIs, an issue that has been the source of considerable debate in the scientific literature [34]. Potential mechanisms include differences in keratin thickness and HIV target cell density between inner and outer foreskin layers; changes in penile microbiome, and/or genital mucosal moisture levels following circumcision [34,35]. Understanding correlates of protection at mucosal level is the focus of our current research [36]. Second, our findings illustrate the importance of evaluating traditional and contemporary practices that have the potential to impact on prevention and care strategies within a wider socio-cultural and epidemiological context. In PNG, providing circumcision services at population level for HIV prevention has not been viewed as cost-effective [37] while the current study suggests that strategies to eliminate other forms of foreskin cutting (as previously proposed in order to reduce the risk of adverse events associated with procedures that are typically carried out in the community) [38] could be counterproductive. Future efforts in PNG should focus primarily on reducing harm that might arise through unsafe cutting practices (e.g. sharing of cutting instruments) and surgical complications (e.g. haemorrhage) [4]. A more problematic issue will be to design an appropriate public health response given the finding that a simple to perform, full dorsal longitudinal foreskin cut conducted before sexual debut may confer significant protection against the acquisition of HIV and other STIs in this setting. The potential public health impact, cost-effectiveness and health system implications of prevention strategies based on the dorsal longitudinal cut also need to be compared with that of medical male circumcision, which importantly, has proven efficacy against HIV acquisition among men who are already sexually active [8–10], unlike the dorsal cut. The process by which these issues are resolved will provide valuable insights for other countries seeking to design and implement culturally appropriate, locally acceptable and scientifically robust HIV/STI prevention strategies.

## Conclusions

In this large cross-sectional study, men with a dorsal longitudinal foreskin cut resulting in the complete exposure of the glans penis were significantly less likely to have HIV, HSV-2 or active syphilis compared with uncut men, supporting our hypothesis that this form of cutting provides protection against the acquisition of these infections in the same manner as male circumcision.

Partial cuts were not associated with lower HIV/STI prevalences. The presence of different types of foreskin cut among a high proportion of men in this setting provides a unique opportunity to investigate the fundamental immunohistologic mechanisms by which male circumcision confers protection against HIV and other STIs.
